# The Outcome of Conversion to Hand-Assisted Laparoscopic Surgery in Laparoscopic Liver Resection

**DOI:** 10.3390/jcm12144808

**Published:** 2023-07-21

**Authors:** Shinichiro Nakada, Yuichiro Otsuka, Jun Ishii, Tetsuya Maeda, Kazutaka Kimura, Yu Matsumoto, Yuko Ito, Hideaki Shimada, Kimihiko Funahashi, Masayuki Ohtsuka, Hironori Kaneko

**Affiliations:** 1 Division of General and Gastroenterological Surgery, Department of Surgery, Toho University Faculty of Medicine, 6-11-1, Omorinishi, Otaku, Tokyo 143-8541, Japan; nakadanakada777@yahoo.co.jp (S.N.); hideaki.shimada@med.toho-u.ac.jp (H.S.); kingkong@med.toho-u.ac.jp (K.F.); hironori@med.toho-u.ac.jp (H.K.); 2 Department of General Surgery, Graduate School of Medicine, Chiba University, Chiba 260-8670, Japan; otsuka-m@faculty.chiba-u.jp

**Keywords:** laparoscopic liver resection, hand-assisted laparoscopic surgery, conversion surgery

## Abstract

Background: Hand-assisted laparoscopic surgery (HALS) is known as a useful option. However, the outcome and predictor of conversion to HALS in laparoscopic liver resection (LLR) are unclear. Methods: Data from consecutive patients who planned pure LLR between 2011 and 2020 were retrospectively reviewed. Univariate and multivariate analyses were performed and compared pure LLR, HALS, and converted open liver resection (OLR). Results: Among the 169 LLRs, conversion to HALS was performed in 19 (11.2%) and conversion to OLR in 16 (9.5%). The most frequent reasons for conversion to HALS were failure to progress (11 cases). Subsequently, bleeding (3 cases), severe adhesion (2 cases), and oncological factors (2 cases) were the reasons. In the multivariable analysis, the tumor located in segments 7 or 8 (*p* = 0.002) was evaluated as a predictor of conversion to HALS. Pure LLR and HALS were associated with less blood loss than conversion to OLR (*p* = 0.005 and *p* = 0.014, respectively). However, there was no significant difference in operation time, hospital stay, or severe complications. Conclusions: The predictor of conversion to HALS was a tumor located in segments 7 or 8. The outcome of conversion to HALS was not inferior to pure LLR in terms of bleeding, operation time, hospital stay, or severe complication.

## 1. Introduction

Laparoscopic liver resection (LLR) is now a useful technique and is increasingly performed worldwide [1,2,3]. The evolution of surgical techniques and instruments used in LLR has allowed the development and advancement of minimally invasive approaches for resectioning hepatic lesions [4,5,6]. Nevertheless, approximately 5–10% of LLRs still require conversion to open liver resection (OLR) due to surgical difficulties, even in high-volume centers or procedures performed by experienced surgeons [7,8,9,10,11]. The most frequent reasons for conversion to open surgery include bleeding, failure to progress, severe adhesions, and oncological concerns [7,8,9,10,11,12]. One of the largest studies on converting LLR to OLR showed that conversion affects surgical outcomes from a multicenter review of 2861 patients [7]. Patients who underwent elective conversion because of unfavorable intraoperative findings, such as failure of progression and severe adhesion, exhibited better outcomes than patients who underwent an emergency conversion secondary to an unfavorable intraoperative event, such as bleeding or oncological concerns during or after resection, in terms of hospital stay, severe complications, and 90-day mortality. The predictors of conversion surgery included major hepatectomy, the presence of a posterosuperior segment, obesity, large tumors, bile duct reconstruction, and previous abdominal surgery [7,8,9,11,12]. Conversion surgery was due to surgical difficulty arising from complications in LLR. The resections were classified as minor, technically major, or anatomically major, and there were statistically significant differences among these groups regarding the likelihood of conversion [13].

Hand-assisted laparoscopic surgery (HALS) is useful in selected cases, especially for large or posterior lesions [14,15,16]. Hand insertion can improve the surgical field and facilitate temporary hemostasis of minor bleeding from the transected surface with finger compression [15,16]. Smaller incisions allow collateral abdominal wall vascularization to be preserved in patients with cirrhosis, diminish postoperative pain, and enhance early rehabilitation [1,8,17]. HALS can also be used to manage intraoperative difficulties and can theoretically decrease the frequency of conversion to a fully open incision [1].

However, the conditions for selecting cases that can be continued with a hand-assisted procedure or require conversion to open surgery are still unclear. Furthermore, the outcome of conversion to HALS remains unclear, even though planned HALS is known to be superior to open surgery regarding bleeding control and postoperative recovery. To the best of our knowledge, few reports have focused on conversion to HALS, and no previous reports have described the predictors of conversion to HALS by using statistical analysis or attempting to predict difficult cases that will require pure LLR. This type of analysis may facilitate patient selection and identification of appropriate indications for LLR. To address this deficit, we aimed to determine the predictors of conversion to HALS or OLR using statistical analysis to assess the outcomes of conversion to HALS.

## 2. Patients and Methods

### 2.1. Study Population

**Figure 1:**
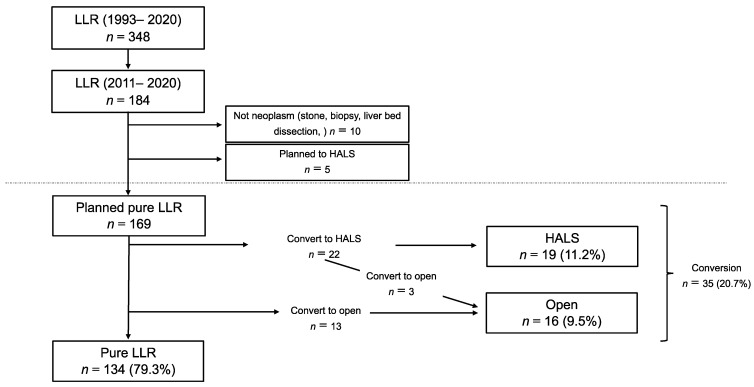
Patient selection: A total of 184 patients were planned with pure LLR. We excluded 15 patients who were not neoplasm or planned HALS. The remaining 169 patients were included in the study.

All consecutive patients who underwent liver resection (LR) at the Toho University Omori Medical Center, Tokyo, Japan, between January 2011 and December 2020 were included. We performed laparoscopic liver surgery on 348 patients between 1993 and 2020. It was reported that the conversion rate significantly decreased after gaining experience from performing 100 LLRs [7]. To reduce the bias caused by early differences in technical proficiency, we chose patients who had undergone the procedures in the last ten years. Data were retrieved from a prospectively maintained database and were analyzed retrospectively. This study included LR for benign and malignant liver tumors. No neoplasms, including stone, biopsy, and liver bed dissection, were excluded (Figure 1). LR was guided by the volume of the future remnant liver, the indocyanine green retention rate, and the probability of achieving curative resection. The indications for LLR were based on oncological and technical terms. The contraindications for LLR included extensive tumor invasions, such as tumor thrombus to the confluence of the major hepatic vein, IVC, or hepatic hilum. It was reported that the proximity of the tumor to the main or second branches of Glisson’s tree, major hepatic vein, and inferior vena cava was significantly correlated with the difficulty of LLR. According to this report, we defined tumor proximity as within 10 mm between the tumor and these vessels [18,19]. The therapeutic strategy and final decision to perform resections or other treatments were discussed during multidisciplinary oncological meetings. The study design was presented to and approved by the Ethics Committee of Toho University Omori Medical Center (ID number M21039 20200 20196 19056 18002). 

### 2.2. Surgical Technique for LLR

Our policy and LLR techniques have been described in detail previously [5,20]. Briefly, patients with a tumor requiring mobilization of the right liver underwent surgery in the left semilateral position, and those with a tumor in the inferior part of the right liver or the left lobe underwent surgery in the supine position. Although the trocar sites differed according to the tumor location, the laparoscope was usually inserted at the peri umbilicus, and three or four trocars for manipulation were placed in a concentric circle radiating from the tumor in nonanatomical partial hepatectomies, while four trocars were placed on the epigastrium, right hypochondrium, and both sides of the abdomen in anatomical hepatectomies. Laparoscopic ultrasonography was performed to confirm the tumor’s location in relation to the vascular anatomy and to identify other liver lesions. For the transection of the liver parenchyma, laparoscopic coagulating shears were used to transect the superficial liver parenchyma. A Cavitron ultrasonic surgical aspirator or clamp-crushing method was used to transect deeper portions of the liver. Small vessels were transected using a bipolar sealing system or by clipping. A stapling device was usually employed to transect a significant diameter of Glisson’s pedicle or hepatic veins. 

### 2.3. Technical Aspects of Conversion to HALS and OLR

According to our policy, conversion to HALS is one of the options to rescue and recover the procedure when difficulties would be encountered if pure LLR were continued. When a case is considered unsafe or presents the risk of oncological curability, we choose conversion to OLR. An additional 7–8 cm incision was required for hand-assisted procedures, corresponding to the surgeon’s hand size. This incision was located slightly away from the tumor and was usually placed in the lower right middle abdomen. When the hand was not being utilized, the incision could be used to insert an additional 12 mm trocar through the Lap-disk^®^ (Hakko-medical, Nagano, Japan). The conversion was defined as needing an additional incision other than incisions for specimen extraction, HALS, or laparotomy during the procedure. Conversion from a pure laparoscopic procedure was decided based on the surgical procedure. The reasons for conversion were categorized according to a previous study: hemorrhage considered difficult to control under laparoscopy, failure to progress, severe adhesions, the operative discovery of unexpected tumor spread, and operative incidents other than hemorrhage [7]. The types of conversion were categorized as either HALS or OLR.

### 2.4. Statistical Analyses

Patient characteristics were reported as mean ± SD, median with IQR or as a percentage, according to the nature of the data. Continuous variables were compared using the Mann–Whitney U test. The cutoff values for continuous factors were determined to be within the normal range. Multivariate analysis of risk factors associated with conversion to HALS or conversion to OLR was performed using logistic regression analysis. A *p*-value < 0.05 on univariate analysis was necessary for entry into the model. Statistical analyses were performed using JMP^®^ Pro 13.0.0 (SAS Institute Inc., Cary, NC, USA).

## 3. Results

### 3.1. Characteristics of the Study Population

LLR was planned for 184 patients between 2011 and 2020. Of these, ten patients did not have liver neoplasms or gallbladder cancer (stone, biopsy, liver bed dissection), and five patients underwent HALS or hybrid surgery because the tumor was large or expected to show severe adhesion. We excluded these 15 patients and retrospectively reviewed the findings for the remaining 169 patients (Figure 1). The baseline characteristics of the patients are presented in Table 1. Briefly, the mean patient age was 66.5 years ± 11.4, and 113 patients (66.9%) were males. The median tumor size was 23 mm (IQR: 15–40). Multiple resections were performed in 22 patients (13.0%), and anatomical resection was performed in 63 patients (37.3%). One hundred-five patients (62.1%) had previously undergone abdominal surgeries, including repeat LR in twenty-three patients (13.6%).

### 3.2. Characteristics of Patients Who Underwent Conversion to HALS or OLR

Among the 169 LLRs, 19 (11.2%) were converted to HALS, and 16 (9.5%) were converted to OLR, including three cases in which conversion to HALS was attempted initially before the final conversion to OLR. Of the 16 patients who underwent OLR, 3 underwent additional small incisions; in one case, this was due to other procedures, and in two, this was due to severe adhesion around the hepatoduodenal ligament. The reasons for these conversions are described in Table 2. Failure to progress was the most common cause of conversion to HALS (11 cases). In all cases, the reason for failure to progress was due to difficulty in acquiring sufficient operative exposure under pure laparoscopic maneuvers. In addition, difficulty controlling bleeding (3 cases), severe adhesion (2 cases), and oncological factors (2 cases) were the reasons for conversion to HALS. The other patient sustained a small bowel injury due to severe adhesion peeling and recovered after a small incision on the abdomen, followed by switching to the HALS technique (1 case).

In contrast, the most frequent reasons for conversion to OLR were oncological concerns (7 cases), strong adhesions (4 cases), bleeding control (3 cases), and the need for further exploration due to common bile duct injury (1 case).

### 3.3. Predictors for Conversion to HALS and Conversion to OLR

The results of univariate and multivariate analyses of conversion HALS are presented in Table 1. In the univariate analysis, male sex, abnormal ICG r-15 test results, and the presence of tumors in segments 7 or 8 were evaluated. We found no significant differences related to body mass index, tumor size, or previous abdominal surgery, including liver resection. Multivariate analysis identified tumor location in segments 7 or 8 (*p* = 0.038) as a predictor. 

For conversion to OLR, the results of univariate and multivariate analyses are presented in Table 3. In the univariate analysis, anatomical LR and tumor proximity to the major vessels were evaluated. While there were no significant differences related to BMI, the abnormal value of the ICG test, tumor size, or previous operation, multivariate analysis identified the tumor proximity to major vessels (*p* = 0.033) as the predictor.

### 3.4. Comparison of Patients Who Underwent Conversion to HALS with Those Who Underwent Pure LLR or Conversion to OLR

**Figure 2:**
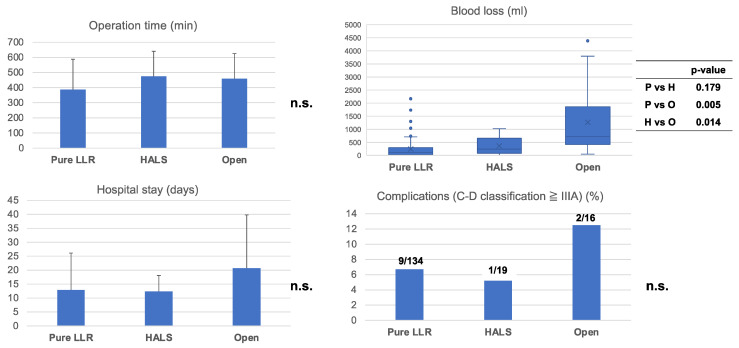
Outcome of pure LLR, conversion HALS or convert to OLR: Comparison of each outcome, pure LLR, conversion to HALS, or conversion to OLR. There was no significant difference in operation time, severe complication (more than Clavien-Dindo classification IIIa), or hospital stay. In terms of blood loss, patients who underwent a conversion to OLR had larger amounts of blood loss more frequently than those who had converted to HALS (*p* = 0.014) or pure LLR (*p* = 0.007). n.s., not significant.

To assess the outcomes of HALS, we compared the results with those obtained after pure LLR and after conversion to OLR (Figure 2). No significant differences were observed in operation time (min) (pure LLR group: 387.2 ± 201.5 min; HALS group: 474.8 ± 170.2 min; and OLR group: 459.1 ± 171.9 min, *p* = n.s.), in-hospital stay (days) (pure LLR group: 12.9 ± 13.3 days; HALS group: 12.4 ± 5.8 days; OLR group: 20.7 ± 19.7 days, *p* = n.s.), or severe complications (more than Clavien–Dindo classification IIIa) (pure LLR group: 9 cases (6.7%); HALS group: 1 case (5.3%): OLR group: 2 cases (12.5%), *p* = n.s.). Patients who underwent conversion to OLR showed a larger amount of blood loss (Median: 720 mL (IQR: 25.5–297.5)) than those who underwent conversion to HALS (Median: 238 mL, (IQR: 85–563) *p* = 0.014) or received pure LLR (Median: 111 mL, (IQR: 25.5–297.5) *p* = 0.005). There was no significant difference between pure LLR and HALS in blood loss (*p* = 0.179).

## 4. Discussion

HALS is known to be beneficial in selected cases. However, the predictors and outcomes of conversion to HALS remain unclear. In our study, tumors in segments 7 or 8 were evaluated as the only predictor of conversion to HALS. In contrast, tumor proximity to major vessels was the predictor of conversion to OLR on multivariate analysis. The outcomes of patients who underwent HALS were not worse than those who underwent pure LLR in terms of bleeding, operation time, hospital stay, and severe complications. In contrast, patients who converted to OLR showed larger bleeding than those who underwent pure LLR or converted to HALS. Thus, in converting surgery, conversion to HALS is a good option to maintain a benefit and overcome the limitations of the pure laparoscopic procedure.

In this study, failure to progress, mainly due to difficulty acquiring sufficient operative exposure, was the most frequent reason for conversion surgery (12 cases of 169). Of these, it was possible to perform HALS in 11 cases (91.7%), thereby avoiding full open surgery. Tumor location in S7 or S8 was evaluated as the only predictor of conversion to HALS. This finding is supported by a previous report that classified resections in the posterosuperior segment as one the most technically challenging resections and found that they were associated with higher conversion rates than resections in the anterolateral segments [21]. Studies of difficulty scores also defined segments 7 and 8 resections as the most difficult [18,19]. Previous reports have shown the usefulness of HALS for posterosuperior lesions due to liver mobilization facilitated by the use of the hand. 

Several studies have reported on the learning curve of LLR. Nomi et al. [22] reported that 45 laparoscopic major hepatectomy procedures were required to reduce operating time. Vigano et al. showed that having adjusted for case-mix, the cumulative sum analysis demonstrated a learning curve for laparoscopic hepatectomies of 60 cases [23]. Though there were no reports discussing the different learning curves of HALS and LLR, hand-assisted surgery can benefit trainees and enhance safety during surgical procedures. It was reported that uncontrolled bleeding is the most frequent reason for converting to open [7,8,9,11,12,13]. We previously reported that HALS is one of the good options for bleeding control [24]. Hand-assisted techniques permit surgeons to obtain sufficient operative exposure and achieve hemostasis by direct compression. Therefore, we believe that the HALS procedure is one of the options for minimally invasive laparoscopic surgery.

In this cohort, 50 patients were treated with surgery in segments 7 or 8. Of them, 13 (26%) and seven patients (14%) needed to convert to HALS or OLR, respectively. The successful rate of pure LLR was 60%. LLR for S7,8 is relatively difficult, but more than half of the cases can be performed with pure LLR. Therefore, it is important to plan for pure LLR while considering the option of HALS conversion when it is difficult. Although there were no statistically significant differences related to abnormal liver function, this parameter tended to predict conversion to HALS (*p* = 0.065). Liver stiffness prevents sufficient liver mobilization using only laparoscopic forceps. An inserted hand can help mobilize the liver [14,15,16]. 

Our results eliminate tumor proximity to a major vessel as a predictor of conversion to OLR. In our study, this term is related and explained by oncological concerns, the most frequent reason for converting to OLR. Previous reports showed that tumor proximity to a major vessel was a factor of difficulty in performing LLR [18,19]. Even using HALS, the oncological situation, such as extensive tumor invasion, including tumor thrombus to the confluence of the major hepatic vein, IVC, or hepatic hilum, are limitations of laparoscopic surgery.

Previous reports have shown that LLR is superior to OLR in selected cases [25,26,27], which can be attributed to reduced postoperative pain and preservation of collateral abdominal wall vascularization. Although pure LLR has been compared with HALS using propensity-matching scores, HALS did not lead to inferior outcomes in previous studies [16,28]. In our study, the outcomes of conversion to HALS were not inferior to those of pure LLR. However, patients who underwent conversion to open surgery showed larger bleeding than those who underwent pure LLR or converted to HALS. Thus, when recovery after conversion to HALS is possible, this approach is worth trying.

Our study had some limitations. First, the sample size was small, and the study was conducted retrospectively using data from a relatively long-term single-center cohort, so certain biases in patient selection could not be avoided (e.g., the indications for LLR, previous therapy, and applied treatment variables). However, this long-term evaluation made it possible to include the variant cases, for example, LLR after an abdominal operation or repeat LR for recurrent tumors. Second, this study focused only on planned pure LR. Therefore, the possibility of selection bias for conditions requiring LLR, such as large tumors, invasion of major vessels, and multiple tumors, could not be ruled out. Thus, future studies that include planned OLR are warranted.

In conclusion, more than half of the cases that required conversion could be converted to HALS, and the outcome of conversion HALS was not inferior to pure LLR in terms of bleeding, operation time, and severe complications. Conversion to HALS is useful for cases with failure of progression and tumors in posterosuperior lesions. However, conversion to OLR was required in cases with oncological concerns.

## Figures and Tables

**Table 1:** Characteristics of all patients, and uni- and multivariate analysis of conversion to HALS.

	Number of Patients (%)	Univariate Analysis			Multivariate Analysis		
		Odds Ratio	95% CI	*p*-Value	Odds Ratio	95% CI	*p*-Value
Preoperative characters of patients							
Age (≥70)	71 (42.0%)	2.04	0.775–5.368	0.149			
Gender (male)	113 (66.9%)	4.781	1.064–21.484	0.041	3.613	0.773–16.897	0.103
BMI (≥25 kg/m^2^)	45 (26.6%)	1.313	0.467–3.694	0.605			
Hepatitis B and/or C (positive)	54 (32.0%)	0.938	0.331–2.654	0.903			
Child–Pugh (class B or C)	9 (5.3%)	0.986	0.116–8.347	0.99			
ICG r-15 (≥10%)	62 (36.7%)	2.669	1.010–7.050	0.048	2.648	0.942–7.442	0.065
Operation period (between 2010–2015)	86 (50.9%)	2.476	0.894–6.861	0.081			
Tumor character and LR							
Large tumor size (≥30 mm)	68 (40.2%)	2.243	0.852–5.909	0.102			
Large tumor size (≥50 mm)	29 (17.2%)	1.333	0.408–4.354	0.634			
Tumor number (≥2)	38 (22.5%)	2.333	0.846–6.435	0.102			
Anatomical LR	63 (37.3%)	0.567	0.194–1.656	0.299			
Located in S7 or 8	50 (29.6%)	5.053	1.855–13.764	0.002	4.948	1.751–13.980	0.002
Multiple liver resection (≥2)	22 (13.0%)	0.811	0.174–3.792	0.79			
Proximity to major vessel	22 (13.0%)	1.293	0.344–4.857	0.704			
Previous operation							
Previous abdominal surgery	105 (62.1%)	1.366	0.492–3.794	0.55			
Previous liver resection	23 (13.6%)	0.722	0.156–3.358	0.679			

BMI, body mass index; LR, liver resection; S7 or 8, Segment 7 or 8.

**Table 2:** Reasons for conversion surgery.

	Convert to HALS (*n* = 19)	Convert to OLR (*n* = 16)
	Number/Probability of Event	Number/Probability of Event
Failure to progress	11/57.8%	1/6.3%
Adhesion	2/10.5%	4/25.0%
Bleeding	3/15.8%	3/18.8%
Oncological concern	2/10.5%	7/43.8%
Need for further processing ^※^	1/5.3%	1/6.3%

HALS, hand-assisted liver surgery; OLR, open liver resection. ^※^ One case required recovery from a small bowel injury and another needed repairing the common bile duct injury.

**Table 3:** Uni- and multivariate analysis of conversion to OLR.

	Univariate Analysis			Multivariate Analysis		
	Odds Ratio	95% CI	*p*-Value	Odds Ratio	95% CI	*p*-Value
Preoperative characters of patients						
Age (≥70)	0.803	0.278–2.322	0.686			
Gender (male)	1.545	0.199–2.107	0.470			
BMI (≥25 kg/m^2^)	0.911	0.278–2.984	0.877			
Hepatitis B and/or C (positive)	1.199	0.372–3.861	0.761			
Child–Pugh (class B or C)	2.980	0.564–15.740	0.199			
ICG r-15 (≥10%)	1.833	0.652–5.159	0.251			
Operation between 2010–2015	0.789	0.280–2.224	0.653			
Tumor character and LR						
Large tumor size (≥30 mm)	0.881	0.304–2.548	0.815			
Large tumor size (≥50 mm)	2.443	0.779–7.665	0.126			
Tumor number (≥2)	1.718	0.557–5.303	0.346			
Anatomical LR	4.273	1.410–12.950	0.010	1.933	0.512–7.304	0.331
Located in S7 or 8	1.990	0.697–5.679	0.199			
Multiple liver resection (≥2)	2.667	0.773–9.204	0.121			
Proximity to major vessel	9.929	3.228–30.538	<0.001	5.795	1.469–22.862	0.004
Previous operation						
Previous abdominal surgery	1.381	0.459–4.174	0.568			
Previous liver resection	0.898	0.190–4.237	0.892			

OLR, open liver resection; BMI, body mass index; LR, liver resection; S7 or 8, Segment 7 or 8.

## Data Availability

The data presented in this study are limitedly available on request to the corresponding author.
